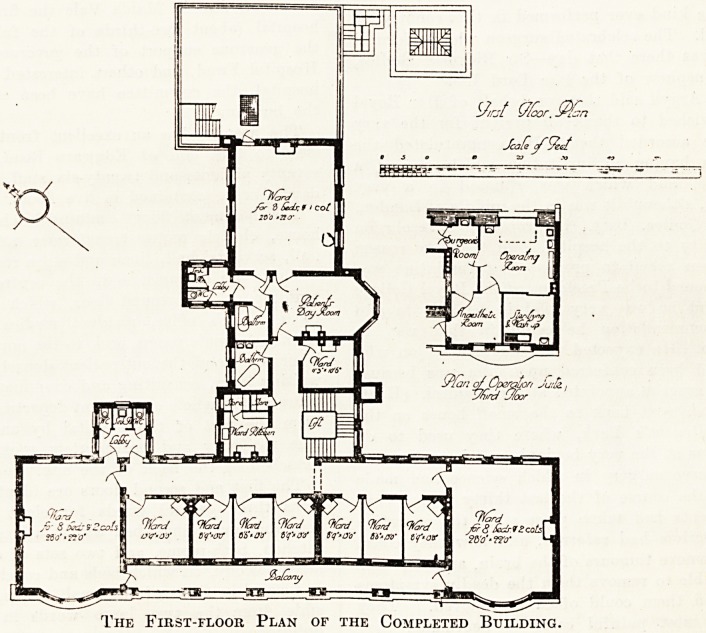# Hospital for Epilepsy and Paralysis

**Published:** 1913-08-09

**Authors:** 


### HOSPITAL FOR EPILEPSY AND PARALYSIS.

### Princess Louise Opens the Completed Building.

Princess Louise, Duchess of Argyll, attended the Hospital for Epilepsy and Paralysis, Maida Vale, W., on Friday, July 25, to open an extension of the hospital, thereby completing the original scheme which the committee set themselves to accomplish when the institution was removed ten years ago from Regent's Park to the present site. The Princess was received by Mr. Charles Drummond (chairman and treasurer) and the members of the committee, by Dr. George Ogilvie (senior physician) and the members of the medical staff, Mr. Keith D. Young, F.R.I.B.A. (the architect), Dr. Hugh Paterson (resident medical officer), Miss Weston (matron), and the Secretary (Mr. H. W. Burleigh). A handsome bouquet of flowers was presented by a little patient, Ena Hawley, to the Princess, which her Royal Highness accepted.

Mr. Charles Drummond, in asking Her Royal Highness to declare the building open, said that as chairman of the committee he had been asked to say a few words in reference to the progress which the hospital had made since the Princess honoured them by a visit ten years ago and declared the first portion open. At the time they felt great anxiety about the future. They found themselves faced with the problem of how they were to raise the largely increased expenditure which the new hospital entailed. The rates and taxes swallowed up the whole of their income from annual subscriptions, and they had to provide for the maintenance of sixteen additional beds, which, of course, meant an increase of expenditure in every department. It seemed almost inevitable that for many years the committee would have to devote all their energies to raising the necessary income to keep the institution going, and that the completion of the buildings would be nothing but a dream of the future which none of them hoped to realise. Happily, however, good fortune still continued to attend them, and they were able year by year to meet their expenses without running into debt. For a long time they had found that the increased accommodation which the hospital gave them was absolutely inadequate to the demands made upon it. The number of applicants for admission increased every year, with the result that they were obliged to keep many urgent cases waiting. The question became so pressing that there was nothing for the committee but to face the situation and to start the completion of the hospital without further delay. The extension would give them twenty-eight additional beds, making seventy in all. The new building had cost £8,000, and the response to their appeal for contributions had been most generous. (Hear, hear.) Only a day or two ago they received a gift of £1,000 from an anonymous benefactor, to whom their most hearty thanks were due. Altogether they had received £5,500, and their past experience led them to hope that the benevolent public would supply the remaining £2,500. On behalf of the committee he (Mr. Drummond) wished to say that they owed a deep debt of gratitude to the secretary and the matron for the way they had worked for the hospital. As to their secretary, he could not pay a higher tribute to his work than was paid by the gentleman who recently visited the hospital on behalf of King Edward's Fund, and who, before leaving, said it had been a genuine pleasure to inspect an institution where the arrangements in every department were so admirably carried out in every detail. (Hear, hear.) It was hoped that the opening of the new wing under such happy auspices would enable them to take in a larger number of patients suffering from

The First-floor Plan of the Completed Building.

maladies of the nervous system which it was their object to relieve.

Her Royal Highness then, amid applause, said : " I have great pleasure in wishing this new building every success on this day of its opening." There followed a short religious service conducted by the Rev. L. J. Percival (rural dean of Marylebone), assisted by the Rev. A. V. Magee (chaplain of the hospital).

Dr. George Ogilvie, in proposing a vote of thanks to Her Royal Highness Princess Louise, said that the hospital was now complete, and even now was certainly not a large one, for it contained not more than seventy-five beds, but, as they said in the French language, " Il y a des bons onguents dans les petites boîtes." Many years ago he remembered a case when a poor patient was admitted suffering from constant and unbearable headaches, convulsive seizures, and progressive blindness, due to pressure from an intracranial tumour. It was deemed advisable by the medical staff to have a surgical operation. Their surgeon was called in, and the result of the operation was cessation of the headaches and relief from all the other attendant troubles. This was the first operation of the kind ever performed in this country, if not in the world. The celebrated surgeon who performed the operation was there that day—Sir Rickman Godlee, P.R.C.S., and nephew of the late Lord Lister.

The Duke of Argyll said that, on behalf of Her Royal Highness, he wished to thank all present for the very kindly welcome accorded them. He congratulated the committee upon having an institution which was of a manageable size, and which was situated in a very healthy position, although it was in the middle of London.

Sir Rickman Godlee, Bart., in proposing a resolution wishing prosperity to the hospital, said that one reason why he had been asked to propose that resolution was because he happened to be President of the Royal College of Surgeons, and he was very glad to be able to add what official commendation he could in that way in addressing them. He expected that another reason why that honour had been conferred upon him was because he was the first surgeon appointed at the hospital. (Hear, hear.) His mind went back to the small house on the north side of Regent's Park, where they used to do various operations at the very beginning of improvements in brain and nerve surgery in which science had made such strides in the course of the last thirty years. Very great improvements had taken place since the operation to which Dr. Ogilvie had referred, and now they could deal with and remove tumours of the brain, and if it was not always possible to remove them the deadly symptoms that accompanied them could often be relieved. They could cure the most painful cases of neuralgia; they were able to fortify nerves that had become paralysed, and they could treat many other nervous disorders which previously they could do nothing for. (Hear, hear.) He most cordially wished prosperity to the hospital from the point of view of the patients who would be admitted into the wards, and there was also the point of view of research in which he hoped the institution would have a prosperous future. Research, of course, could be carried on in private houses, but nowhere so well as in a hospital, where, besides the wards and the operating theatre, they had a laboratory. He was told that a pathological laboratory had been built in that hospital. It was difficult if not impossible to support a laboratory and a pathologist out of the funds that were subscribed by a generous public for the relief of sickness, and therefore he would urge upon any person present when the time comes for loosening their purse strings to remember that the endowment of research in the form of a laboratory in a hospital like that was a very important matter.

The Mayor of Marylebone seconded the resolution. One reason especially which appealed to him in connection with the hospital was that it was doing a splendid work in encouraging persons to voluntarily pay something towards the cost of their treatment. The resolution was carried with acclamation, and briefly responded to by Mr. G. M. Bauer, a member of the committee.

Before leaving, the Royal party were conducted over the hospital by Mr. Keith Young, the architect. The official description of the buildings we append below.

### Architect's Description.

The authorities of this institution are to be congratulated on providing a new and up-to-date building in which to carry on their beneficent work. Ten years ago the lease of the old building in Portland Terrace, Regent's Park, fell in, and renewal could not be obtained, the trustees of the estate having other designs for the site. In spite of tremendous difficulties the committee raised on a new site in Maida Vale the first part of a model hospital (about two-thirds of the full scheme), and by the generous support of the governors, King Edward's Hospital Fund, and others interested in the work of the hospital, the committee have been enabled to complete the building.

The hospital has an excellent frontage in Maida Vale close to that end of Edgware Road, and provides for seventy patients and twenty-six staff, the total accommodation being contained in five floors.

The basement floor contains kitchen, offices, boiler-house, electric motor transformer room, nurses' dining hall, servants' hall, linen and store rooms.

The main entrance is in the centre of the completed building on the ground floor, which contains the board room, clerk's office, resident medical officers' suite of rooms, matron's rooms, and, to the north, the pathological laboratory, and the out-patient department, consisting of waiting halls, consulting and examination rooms and dispensary, the whole out-patient department being separated from the rest of the hospital by an open-air corridor. The mortuary and post-mortem room stand completely detached at the back of the site.

The first and second floors are identical, and each contains three general wards for eight patients, and two cots and seven single-bed wards, a large day room, ward-kitchen, bath-rooms, and two sets of sanitary offices. A large balcony, on which beds and couches can be wheeled, is formed in front of the small wards, and is also accessible from the two large wards in the front of the building.

The top floor is divided into rooms for the nursing and domestic staffs (there being thirteen single bedrooms for matron, sisters, and nurses, and eight for maids), and the operation suite. This comprises the theatre, anæsthetising, sterilising, and surgeons' rooms. There is a hydraulic lift (of sufficient capacity to accommodate a bed) available from the basement to the top floor. The building is heated by hot water, and the kitchen is installed with gas-cooking apparatus.

Special features of the hospital are its accommodation for rest-cure patients (there being fourteen single-bed wards for such patients, who are free or paying), and its school of massage and electrical treatment, to which the nurses are admitted free and others on payment of small fees.

The total cost of the building has been about £35,000. The builders for the new wing are Messrs. J. W. Falkner and Son.

## Figures and Tables

**Figure f1:**